# Development and initial testing of a new instrument to measure the experience of eczema control in adults and children: Recap of atopic eczema (RECAP)†

**DOI:** 10.1111/bjd.18780

**Published:** 2020-02-20

**Authors:** L.M. Howells, J.R. Chalmers, S. Gran, A. Ahmed, C. Apfelbacher, T. Burton, L. Howie, S. Lawton, M.J. Ridd, N.K. Rogers, A.V. Sears, P. Spuls, L. von Kobyletzki, K.S. Thomas

**Affiliations:** ^1^ Centre of Evidence Based Dermatology University of Nottingham Nottingham UK; ^2^ Patient representative Nottingham UK; ^3^ Department of Medical Sociology Institute of Epidemiology and Preventive Medicine University of Regensburg Regensburg Germany; ^4^ Institute of Social Medicine and Health Economics Otto von Guericke University Magdeburg Magdeburg Germany; ^5^ Patient representative Brisbane Australia; ^6^ Rotherham NHS Foundation Trust Rotherham UK; ^7^ Department of Population Health Science University of Bristol Bristol UK; ^8^ St John's Institute of Dermatology and Peter Gorer Department of Immunobiology School of Immunology and Microbial Sciences King's College London London UK; ^9^ Department of Dermatology Amsterdam University Medical Centers, Academic Medical Center University of Amsterdam Amsterdam Public Health, Infection and Immunity Amsterdam the Netherlands; ^10^ Centre for Clinical Research, Malmö, Lund University Sweden; ^11^ Centre for Clinical Research Örebro University Sweden

## Abstract

**Background:**

Eczema control has been identified as an important outcome by key stakeholders in eczema research (including patients, carers, healthcare professionals and researchers) but no validated instruments for the domain have been identified.

**Objectives:**

To develop a measurement instrument to capture a patient's perspective of eczema control that is suitable for use in eczema clinical trials.

**Methods:**

Best practice for the development of a patient‐reported outcome was followed. A mixed‐methods approach was used to develop and refine a conceptual framework, generate, refine and select items and to test the distribution and construct validity of the final scale. The mixed‐methods approach involved expert panel meetings (including patient representatives, healthcare professionals and methodologists), and data collection using a focus group, cognitive interviews and an online survey with people with eczema and caregivers. Multivariable linear regression was used in the item selection process.

**Results:**

Fourteen expert panel members co‐produced the instrument, with input from people with eczema and caregivers via a focus group (*n* = 6), cognitive interviews (*n* = 13) and an online survey (*n* = 330). The resulting instrument, Recap of atopic eczema (RECAP), is a seven‐item questionnaire that captures eczema control via self or caregiver report. The development process aimed to ensure good content validity and feasibility. Initial testing suggested no floor or ceiling effects and good construct validity. Hypothesized correlation with the Patient‐Oriented Eczema Measure was confirmed [*r*(258) = 0·83, *P* < 0·001].

**Conclusions:**

RECAP has the potential to improve reporting of eczema control in research and clinical practice. Further exploration of measurement properties is required.

**Linked Comment:** Pattinson and Bundy. *Br J Dermatol* 2020; **183**:418–419.

**What's already known about this topic?**

- Eczema control has been identified as an important outcome by key stakeholders in eczema research (including patients, carers, healthcare professionals and researchers).
- Qualitative studies suggest eczema control is a multifaceted and individual experience and no instrument has been identified that captures eczema control in this way.

**What does this study add?**

- We have developed Recap of atopic eczema (RECAP), a seven‐item questionnaire to capture the experience of eczema control in all ages and eczema severities; there are two versions: a self‐reported version for adults and older children with eczema, and a caregiver‐reported version for younger children with eczema.
- Designed with input from people with eczema, caregivers and healthcare professionals to ensure good content validity.
- Initial testing of score distributions and construct validity suggests good measurement properties.

**What are the clinical implications of the work?**

- The RECAP instrument is appropriate and feasible for measuring eczema control in clinical trials and may also be useful in routine practice.

## Abstract 2

**Linked Comment:** Pattinson and Bundy. *Br J Dermatol* 2020; **183**:418–419.

Plain language summary available online

Atopic eczema (syn. eczema, atopic dermatitis) is a common, chronic condition that is characterized by itchy, dry skin that can become cracked and sore and often has a relapsing and remitting disease course. The Harmonising Outcome Measures in Eczema (HOME) initiative recommends ‘long‐term control of eczema’ as a core outcome domain that should be measured in every clinical trial over 3 months’ duration, indicating that it is an important outcome for a range of stakeholders (including patients, carers, healthcare professionals and researchers).^1^ Consensus voting at the HOME V meeting in June 2017 identified the need for a patient global assessment of eczema control.^2^

Qualitative research involving people with eczema, their caregivers and healthcare professionals suggests that eczema control is a multifaceted construct involving changes in disease activity, the treatment and management of the condition and psychological, social and physical functioning.^3^, ^4^ Measuring such a complex construct over time can be challenging, but instruments to capture long‐term control have been developed in other chronic diseases such as asthma and urticaria.^5^, ^6^

This study aimed to develop a new outcome measurement instrument to capture a patient's perspective of eczema control for use in research and clinical practice. The study objectives were to: (i) develop an instrument to capture eczema control that is suitable for use in both adults and children with eczema and (ii) conduct preliminary validation of the new instrument (including assessment of floor and ceiling effects and construct validity).

## Methods

### Study design

**Figure 1:**
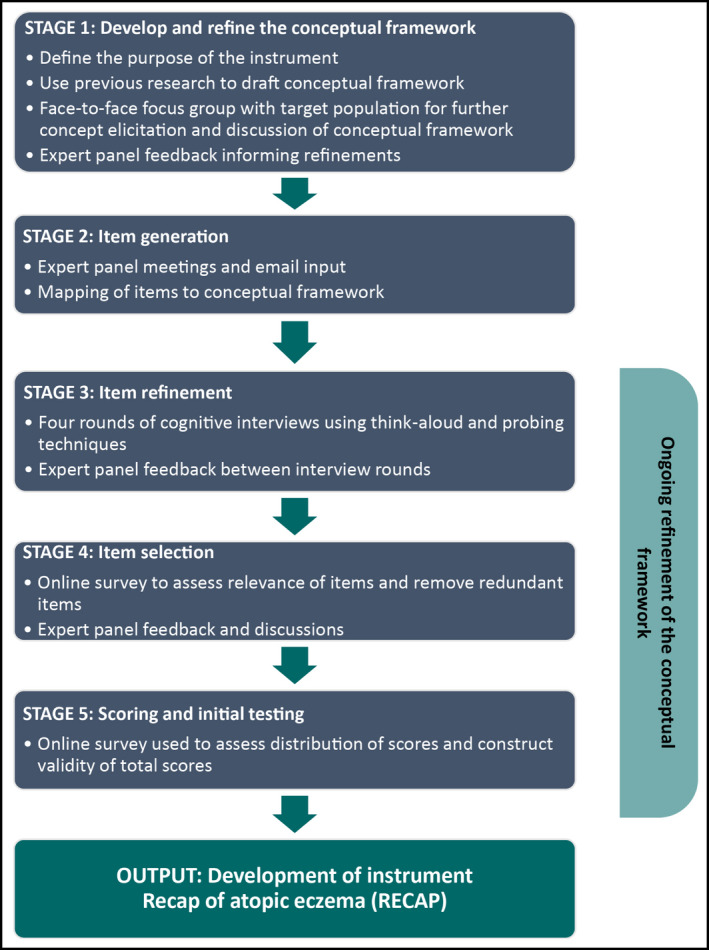
Study design for developing Recap of atopic eczema (RECAP).

This mixed‐methods study included five stages of instrument development as summarized in Figure 1. Methodological guidance for instrument development was followed.^7^, ^8^, ^9^, ^10^, ^11^ The development process was guided by an international expert panel consisting of three dermatologists, a dermatology nurse, a general practitioner, two adults with eczema, two caregivers of children with eczema, four methodologists and a psychologist. Five countries (UK, Germany, Sweden, the Netherlands and Australia) were represented on the expert panel. This project has been approved by the University of Nottingham's Faculty of Medicine & Health Sciences Research Ethics Committee (Refs: 18‐1805 and F14062016 SoM ROD). Both the protocol and data analysis plan were uploaded to the registration portal of the Centre of Evidence Based Dermatology (CEBD) a priori and can be referred to for further methodological details of the study (https://www.nottingham.ac.uk/research/groups/cebd/resources/protocol-registration.aspx).

### Stages of instrument development

#### Stage 1: develop and refine the conceptual framework

**Figure 2:**
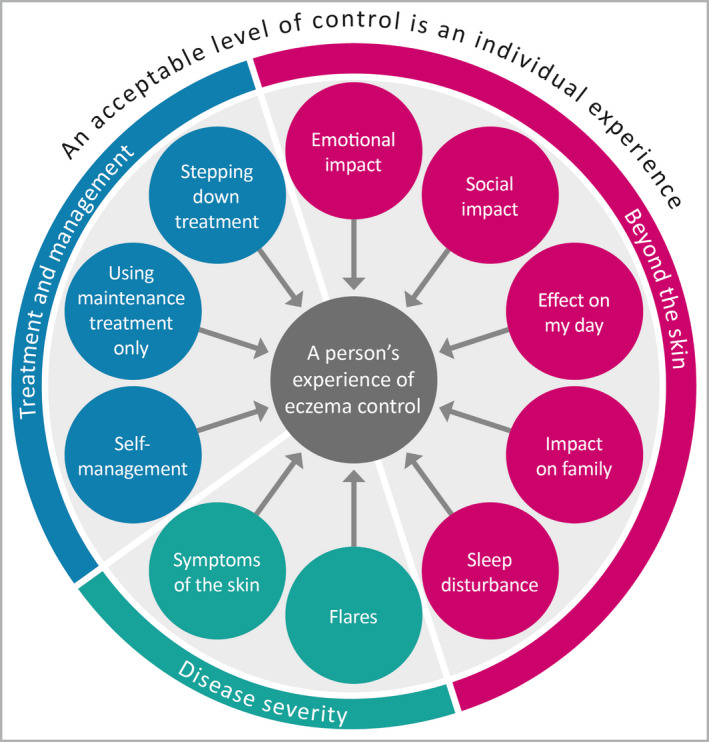
Initial conceptual framework discussed at focus group.

Table 1 outlines the intended purpose of the instrument. The qualitative studies and HOME V meeting discussions suggest that the focus of this instrument should be individual ‘perceptions’ of eczema control. It also is worth noting that this construct as defined is not related to perceptions about the ‘controllability of eczema’, which might relate to what an individual thinks they can do, or what their treatment can do, to control their eczema, but about the attainment of control perceived when reflecting on their experience. The details of what are ‘indicators of control’ are presented in the conceptual framework (Fig. 2). The conceptual framework was drafted by L.M.H., J.R.C., C.A. and K.S.T. through synthesizing findings from an international qualitative study,^3^, ^4^ an international patient survey^12^ and a systematic literature review^13^ relating to the construct of interest.

**Table 1:** Defining the purpose of the instrument (Stage 1)

Intended purpose	Decision
Intended construct of interest	The experience of eczema control. This was defined in this study as ‘the extent to which the various manifestations of eczema and the impact that these have for an individual are removed or meaningfully reduced’
Intended target population	Individuals with eczema of all ages. However, for younger children who do not have the cognitive abilities to answer the questionnaire alone, it is intended that the information will be provided by caregivers or with the assistance of caregivers. The questionnaire is not intended to be exclusive to use in a single disease severity, disease duration, sex or ethnicity
Intended context for use	Primarily designed for use in clinical trials assessing any type of intervention in people with eczema. As a secondary aim, it was also anticipated that the instrument should be appropriate for use in clinical settings

A 2‐h face‐to‐face focus group involving people with eczema and caregivers of children with eczema was conducted to confirm the conceptual framework and to ensure that key items had not been overlooked. This focus group was moderated by C.A., who has experience in moderating groups and training in qualitative research. The focus group followed a topic guide that included open discussion about the construct of interest, followed by discussion focused on the conceptual framework (Appendix S1; see Supporting information). The focus group was recorded and transcribed verbatim. The findings from the focus group were discussed by the expert panel who used this conceptual framework as a starting point to begin item design. The conceptual framework developed suggests this instrument requires a formative measurement model, which has impacted the methodology choices later in the development process.

#### Stage 2: item generation

Driven by the conceptual framework and a short guidance document on constructing questions the expert panel members submitted ideas for items to include in the instrument.^9^, ^14^ The items were then categorized and discussed by the panel. The items were either discarded, kept or amended to produce an initial working list of items.

#### Stage 3: item refinement

Cognitive interviews, which used a range of think‐aloud and probing techniques, were used with the aim to improve the comprehension, comprehensibility and relevance of the questionnaire. The target population was adults (16+ years) with eczema or caregivers of children with eczema living in the UK. Children under 16 years of age could take part if their caregiver was present. Participants were recruited using existing mailing lists held at the CEBD in Nottingham of people interested in eczema‐related research and through social media. All participants had to be proficient in English. There was no exclusion of participants based on age, eczema severity, sex or ethnicity and purposeful sampling aimed to achieve a diverse range of participants based on these characteristics.

Cognitive interviews lasting approximately 1 h each took place either face to face, on the telephone, or via video call depending on participant preference. All interviews were conducted by L.M.H., who has experience and training in qualitative research and the team received further support from a methodological advisor (Dr Paul Leighton, P.L.). Interviews followed a semi‐structured interview guide (Appendix S2; see Supporting information). The participants were asked to answer the items using think‐aloud methods.^15^ The interviewer planned breaks to summarize and use pre‐planned probes to encourage elaboration by participants. Once the think‐aloud process was applied to all items, the interviewer then probed the participants about the items as a global set. Interviews took place in rounds, with the expert panel refining the content in between rounds, and subsequent rounds assessing if the changes had addressed the initial problems. It was planned that rounds would be continued until no further refinements were required. All interviews were recorded and transcribed verbatim.

#### Stage 4: item selection

An online survey was used to conduct an impact analysis, which uses information about frequency of occurrence and the importance of the experiences to assess the relevance of each item. The aim of impact analysis is to assess the importance of each item, as formative models require that the most important items are represented. The survey was also used to conduct a backward stepwise regression analysis. The aim of regression analysis is to reduce the number of items and ensure that each item adds unique information about the experience of eczema control. The target population was the same as for Stage 3, as were the recruitment methods, although the latter was supplemented with the use of posters in various public settings (shops, cafes, libraries, universities, healthcare centres).

Variables included in the survey were age, sex, ethnicity, ‘bother caused by the eczema’, global eczema severity, the Patient‐Oriented Eczema Measure (POEM), and items being considered for inclusion following the item refinement stage. Participants also indicated if that experience had occurred for them/their child over the past year and how important this experience is when thinking about their/their child's eczema.

#### Stage 5: instrument scoring and preliminary validation

The online survey described in Stage 4 was also used to collect data on the final items chosen for inclusion so that these could be scored and tested. Overall scores for the final items were generated using scoring rules determined by the expert panel. These scores were then tested for distribution of scores. The aim was to ensure that the items did not produce a score with a floor or ceiling effect, which is where a high proportion of the total population has a score at the lower or upper end of the scale, respectively.^7^ They were also tested for construct validity, which aims to assess the degree to which the scores of the instrument are consistent with hypotheses that are based on the assumption that the instrument validly measures the construct to be measured.^16^

### Analysis

#### Focus group for Stage 1: develop and refine the conceptual model

Experiences of eczema control were mapped onto the theoretical framework used in previous concept elicitation studies.^3^, ^4^ L.M.H. and P.L. independently coded the data and met to discuss any discrepancies in coding. A qualitative descriptive approach was used to analyse the participants’ responses to the conceptual framework.^17^

#### Cognitive interviews for Stage 3: item refinement

Data were analysed using a top‐down coding approach, and refined with inductive coding (Table S1; see Supporting information). All data were analysed by L.M.H., with secondary coding on selected transcripts by J.R.C. or A.V.S. As it was not possible with the resources available for all transcripts to be coded, transcripts for secondary coding were selected to include both early and final interviews, interviews containing a variety of problems across the coding framework, and selected transcripts with difficult‐to‐code problems. Discrepancies in coding were discussed and resolved via discussion. S.G., K.S.T., L.M.H. and J.R.C. were all involved in ongoing discussions about the coding and between each round of interviews; any problems that were identified and potential solutions were fed back to the expert panel for their input. Based on a saturation model, interview rounds continued to test if problems from previous interview rounds were resolved until the authors were satisfied that there were no major problems that could be resolved via further cognitive interviewing. The final round tested items in the final wording that was then used in the online survey for further item reduction.

#### Online survey for Stages 4 and 5: item selection, instrument scoring and preliminary validation

Data were analysed using Stata 15.

*Impact analysis*. For each item concept, the proportion of individuals who had experienced that in the past year was multiplied by the mean score of the importance rating (1, not important; 5, extremely important) to give an impact score ranging from 0 to 5. Whole‐sample analysis and subgroup analysis by age of person with eczema (0–4 years, 5–15 years, 16+ years) were decided a priori. It was predefined that an impact score of less than 2 in any group analysed indicated that an item should not be considered for inclusion in the instrument, as was used in the development of the Urticaria Control Test.^5^

*Multivariable regression analysis*. The potential items were entered as independent variables into multivariable linear regression models. The dependent variable was ‘bother caused by eczema’ (0–10 points). This dependent variable was chosen because there was no ‘gold standard’ measure of ‘eczema control’ that could be used. ‘Bother caused by the eczema’ was agreed by the expert panel to be the most closely aligned measure available (it has been used in previous eczema research) with the concept of ‘eczema control’ as defined for the development of Recap of atopic eczema (RECAP). Sample size was calculated as at least 10 cases per independent variable. The backward elimination variable selection technique was used to determine which items remained in the model. The stopping criteria for this process was *P* = 0·157, which is recommended for sample sizes with between 10 and 25 events per parameter.^18^ The assumptions of multicollinearity, linearity, normality of residuals and homoscedasticity of residuals were met.

*Scoring*. The expert panel agreed scoring rules resulted in all RECAP items being scored from 0 to 4 and weighted equally and added together (total scores ranging from 0 to 28), with a higher score indicating less eczema control.

*Distribution of scores*. Assessment of histograms. A floor or ceiling effect was defined prior to data collection as more than 15% of participants achieving the highest or lowest possible score.^19^

*Construct validity*. Convergent validity assesses if instruments that are theoretically measuring similar constructs are related. POEM measures the construct of ‘eczema‐related morbidity’ by monitoring eczema symptoms over the last week. A systematic review that included studies looking at the measurement properties of POEM concluded that there was limited evidence for good internal consistency, moderate evidence for good construct validity, good responsiveness and good content validity, and unclear evidence of test–retest reliability and measurement error.^20^, ^21^, ^22^, ^23^ Interpretation of POEM has been assessed in the form of the minimally important change and severity bandings.^21^, ^24^, ^25^, ^26^ Pearson's correlation coefficients were used to assess the relationship between POEM (measuring patient‐reported symptoms) and the newly developed instrument. It was hypothesized that correlations would be at least 0·3 (moderately correlated). Discriminative validity is where a measurement instrument is able to distinguish between subgroups of patients. This can be done by comparing the mean scores on the measurement instrument for the subgroups.^7^ Subgroups of participants were categorized based on scores from a global eczema severity measure and POEM severity categories.^25^ It was hypothesized that there would be a linear trend of higher mean RECAP scores for each subgroup of participants categorized with more severe eczema on the global eczema severity measure and the POEM severity categories.

### Patient and public involvement

As expert panel members, two patients and two caregivers took part in face‐to‐face/video meetings, teleconferences and email feedback to co‐design items and input into decisions throughout the process. All reviewed the wording and design of materials (including information sheets, consent forms, advertisement/posters, the online survey). N.K.R. and T.B. piloted the cognitive interview process to give the interviewer feedback on the process. The CEBD Patient Panel day (a patient and public involvement day at the University of Nottingham) provided input during the item refinement process with some key, targeted queries to aid finding solutions to a problem exposed in the cognitive interviews.

## Results

### Participant characteristics

Details of the number of participants, sex and self‐reported ethnicity (where available) are reported for the focus group (Stage 1) and the cognitive interviews (Stage 3) in Table 2. Self‐reported eczema severity ranged from mild to very severe in both the focus group and the cognitive interviews. For the interviews, the age of adults with eczema ranged from 37 to 64 years and all reported onset of eczema as young children. The age of the children of the caregivers taking part ranged from 2 years and 8 months to 14 years. Onset of eczema was reported from 8 weeks old to 2 years and 6 months old. A total of 330 took part in the online survey (Stages 4 and 5), but six of these participants completed only demographic variables. Table 3 provides the participant characteristics for the online survey.

**Table 2:** Focus group and cognitive interview participant characteristics

Participant characteristics	Focus group, *n*	Cognitive interviews, *n*
Total *N*	6	13
*Adults*	5	8
Sex		
Male	2	0
Female	3	8
Ethnicity^a^		
White‐British		5
White‐Scottish		2
Sikh		1
*Caregivers*	1^b^	
Child's sex		
Male	1	3
Female	0	2
Child's ethnicity^a^		
White‐British		5
Welsh/Maltese		1

^a^Ethnicity stated have been preserved as the participants reported. ^b^Although only one person took part primarily as a caregiver, there were an additional two participants who are classified as adults with eczema in this table, but they also had experiences of caring for their children with eczema.

**Table 3:** Online survey participant characteristics

	*n*	%	Mean (± SD)	Range
Age (years)	324	–	22·71	0–66
Under 5^a^	62	19·1	–	–
5–15^a^	77	23·8	–	–
16+	185	57·1	–	–
Sex	324	–	–	–
Male	110	33·95	–	–
Female	211	65·12	–	–
Nonbinary	2	0·62	–	–
Rather not say	1	0·31	–	–
Ethnicity	322	–	–	–
White	300	93·17	–	–
Bangladeshi	1	0·31	–	–
Black Caribbean	2	0·62	–	–
Chinese	6	1·86	–	–
Indian	6	1·86	–	–
Mixed race	5	1·55	–	–
Other Asian (non‐Chinese)	1	0·31	–	–
Sikh	1	0·31	–	–
Total POEM score	263	–	15·12 ± 7·37	0–28
POEM severity banding	263	–	–	–
Clear/almost clear	13	4·9	–	–
Mild	33	12·5	–	–
Moderate	95	36·1	–	–
Severe	96	36·5	–	–
Very severe	26	9·9	–	–
Global severity	266	–	–	–
Clear	6	2·3	–	–
Almost clear	34	12·8	–	–
Mild	65	24·4	–	–
Moderate	121	45·5	–	–
Severe	40	15·0	–	–
Bother caused by the eczema^b^	324	–	5·65 ± 2·56	0–10

^a^For under 16‐year‐olds the survey was completed by a caregiver in 95% of cases (*n* = 132). ^b^How much bother has your/your child's eczema been over the past week? Responses from 0 (no bother at all) to 10 (as much bother as you can imagine)

POEM, Patient‐Oriented Eczema Measure.

### Key stages of instrument development

#### Stage 1: develop and refine the conceptual framework

**Figure 3:**
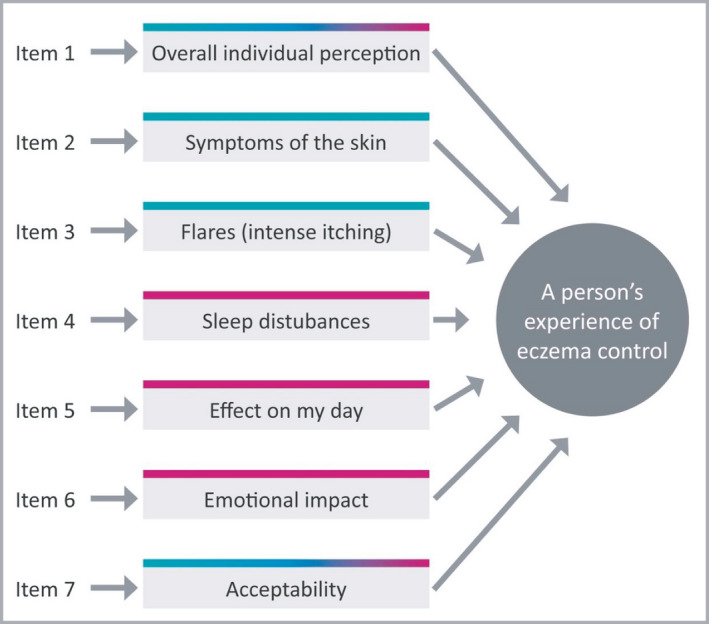
Final conceptual framework. Note. The direction of the arrows indicates that a formative measurement model is most appropriate to use.

Figure 2 shows the initial conceptual framework that was presented to members of the focus group (although more detail was included relating to each concept). Mapping to the thematic framework indicated that data saturation for concept elicitation was reached. Participants confirmed that the framework represented an accurate model of ‘eczema control’ and that the conceptual framework was comprehensive. Nevertheless, some minor refinements were suggested, as summarized in Table 4. The final conceptual framework for the RECAP instrument, after refinement based on all stages of instrument design is presented in Figure 3. The conceptual framework suggests that a formative model is the best approach to developing the measurement model for this construct of interest as multiple unique factors are relevant to the experience, which, when combined together, form the latent variable.^7^

**Table 4:** Refinements to the conceptual framework

Refinement of conceptual framework	Reasons why
Addition of concept predictability of eczema	Participants at the focus group expressed a concern that the predictability of eczema, which related to eczema control in their perception, was not included in the conceptual framework.
Removal of item on predictability	The cognitive interviews suggested that an item asking directly about the predictability of the eczema was not interpreted in line with the construct of interest. It may be that this concept is a related but distinct outcome to be measured.
Removal of concept impact on family	The expert panel meeting led to discussions about designing items on the impact on family and it was felt strongly among stakeholders including patients that this concept was not universal to all. It was also suggested to be a related but distinct construct.
Removal of treatment and management concepts	The cognitive interviews revealed issues regarding the applicability and relevance of treatment items. The expert panel discussed these findings and reviewed the inclusion of these concepts within the framework. Some members wanted these concepts to remain, while others felt they were not part of the construct of interest. The discussions at the HOME V consensus meeting were also referred to, which indicated that stakeholders did not think treatment‐related items were feasible in all clinical trials. Issues that were considered when making this decision: Treatment‐ and management‐related questions are answered differently depending on disease severity and type of treatment used. For example, only people with more severe eczema will have access to systemic therapies. There is difficulty in distinguishing between answers that relate to eczema control and answers that relate to personal choice (e.g. a patient who does not want to use a particular treatment but has low level of control may answer in a way that appears congruent with good control). In many clinical trial situations it is not always possible for patients to change or step up/step down their treatment so these concepts are not always applicable, but were one of the main features of understanding level of control in a nontrial setting by stakeholders who inputted into the conceptual model. The online survey revealed that the items regarding social impacts were not applicable and relevant to young children. This finding was discussed among the expert panel who approved removing this concept.
Change in way ‘overall individual perception’ and ‘acceptability’ were included in the framework	In the initial conceptual model, ‘an acceptable level of control is an individual experience’ was an overarching concept that was considered important, but it was not initially clear how this fit within the design of the instrument. Through expert panel discussions when interpreting the findings from the face‐to‐face focus group and designing items, it was acknowledged that items about the ‘acceptability of eczema’ to an individual and the individual's personal overall perception of ‘how the eczema had been’ were unique perceptions about the experience of eczema control that could be included as items in the measure.

HOME, Harmonising Outcome Measures for Eczema.

#### Stage 2: item generation

Fourteen expert panel members were each asked to submit questions that could be used to capture the key elements of eczema control as outlined in the conceptual framework. This process resulted in an initial list of 154 ideas, although many of the ideas gave multiple alternate options to capture the same concept. Using these as a starting point, the expert panel worked together to group, discard and amend items and make key decisions on how to present the items (e.g. number of response options). On the basis of this discussion, the lead researcher compiled a list of 25 items that were then revised and approved by the expert panel to be tested in the next phase of the development process.

#### Stage 3: item refinement

Thirteen interviews took place over four rounds (round 1, *n* = 5; round 2, *n* = 3; round 3, *n* = 4; and round 4, *n* = 1). The changes that took place over four rounds of cognitive interviews were: the recall period was changed from 4 weeks to 1 week; the number of response options was increased from four to five; the items were changed from statements to questions; the wording was changed to provide clarity; and the language was amended to reflect terms respondents felt had greater resonance and that increased the confidence of respondents in their ability to answer the questions (Table S2; see Supporting information). By the end of the interviews 15 items remained for further testing. Detailed results of this analysis are presented in Appendix S3 (see Supporting information). However, only 14 items were included in subsequent analysis as the expert panel made the decision to remove the remaining treatment‐related item, having reflected on the HOME V meeting decisions about the feasibility of treatment‐related measures, the cognitive interview findings and the conceptual framework refinements.

#### Stage 4: item selection

*Impact analysis*. Data on frequency, importance and impact scores from the online survey are presented in Table 5. ‘Feeling self‐conscious’ scored less than 2 in the age group 0–4 years and ‘feeling isolated’ scored less than 2 across all ages, in the age group 0–4 years and the age group 16+ years. It was predefined that any item with an impact score of < 2 for any of our target groups was considered not relevant and therefore these two items were excluded from the subsequent regression analysis. Items on the ‘acceptability’, ‘overall individual perception’ and ‘treatment been enough’ were not included in the impact analysis due to the expert panel appraising that it was not appropriate to assess the frequency and importance of these items.

**Table 5:** Results of impact analysis^a^

Age group (years)	Frequency (proportion)				Importance (mean score)				Impact score (frequency ×importance)			
	All	0–4	5–15	16+	All	0–4	5–15	16+	All	0–4	5–15	16+
Itchy skin	1	1	1	1	4·77	4·89	4·82	4·7	4·77	4·89	4·82	4·70
Flare	0·9963	1	1	0·9935	4·6	4·81	4·64	4·5	4·58	4·81	4·64	4·47
Had any symptoms	0·9963	1	1	0·9935	4·57	4·63	4·54	4·55	4·55	4·63	4·54	4·52
Skin painful or sore	0·9925	1	0·965	1	4·63	4·74	4·73	4·56	4·60	4·74	4·56	4·56
Intensely itchy skin	0·9736	0·9811	0·9649	0·9742	4·55	4·74	4·66	4·45	4·43	4·65	4·50	4·34
Unable to stop scratching	0·9586	0·9444	0·9474	0·9677	4·58	4·7	4·79	4·46	4·39	4·44	4·54	4·32
Eczema affecting how been feeling	0·937	0·9259	0·9298	0·941	4·4	4·5	4·59	4·29	4·12	4·17	4·27	4·04
Disturbed sleep	0·9023	0·9259	0·9483	0·8766	4·24	4·35	4·81	3·99	3·83	4·03	4·56	3·50
Eczema getting in the way of day‐to‐day activities	0·8647	0·8148	0·8966	0·8701	4·21	4·22	4·47	4·12	3·64	3·44	4·01	3·58
Stopped from doing something wanted or needed to do	0·7895	0·7222	0·8621	0·7806	4·14	4·17	4·38	4·04	3·27	3·01	3·78	3·15
Feeling self‐conscious or embarrassed	0·7857	0·2778	0·8596	0·9355	4·3	3·61	4·59	4·39	3·38	1·00^b^	3·95	4·11
Feeling isolated	0·4906	0·1852	0·569	0·5677	3·5	3·54	4·28	3·15	1·72^b^	0·66^b^	2·44	1·79^b^

^a^Items on the ‘acceptability, ‘overall individual perception’, and ‘treatment been enough’ were not considered appropriate for inclusion in the impact analysis. ^b^An impact score of < 2 was defined a priori as indicating an experience was not relevant to include in the multivariable linear regression analysis.

*Multivariable linear regression analysis*. Two models were developed. The first model contained all 12 items that were still under consideration for inclusion in the final set of items as predictor variables. ‘Bother caused by the eczema’ was used as the outcome variable. Five predictor variables were removed from the model following a backward elimination item reduction technique with a stopping criterion of *P* = 0·157. These included items ‘being unable to stop scratching’ (*P* = 0·809), ‘stopped from doing something wanted or needed to do’ (*P* = 0·438), ‘having flares’ (*P* = 0·314), ‘having any symptoms’ (*P* = 0·809) and ‘painful or sore skin’ (*P* = 0·612). The results of the regression indicated that the seven remaining predictor variables explained 71·1% of the variance in ‘bother caused by the eczema’, *R* ^*2*^ = 0·718, adjusted *R* ^*2*^ = 0·711, *F*(7, 256) = 93·19, *P* < 0·001. Table 6 shows the predictor variables that remained in the model.

**Table 6:** Model 1 Final output (all items included in the final RECAP instrument), *n* = 264

Predictor variables	β	*P*‐value	95% CI
Acceptability of eczema	0·30	0·017	0·05–0·55
Itchy skin	0·19	0·053	–0·002 to 0·38
Sleep disturbance	0·14	0·127	–0·04 to 0·32
Getting in the way of day‐to‐day activities	0·32	0·01	0·08–0·55
Affecting how been feeling	0·13	0·102	–0·03 to 0·29
Intensely itchy skin	0·22	0·009	0·06–0·39
Global	0·92	> 0·001	0·71–1·24

CI, confidence interval; RECAP, Recap of atopic eczema.

**Figure 4:**
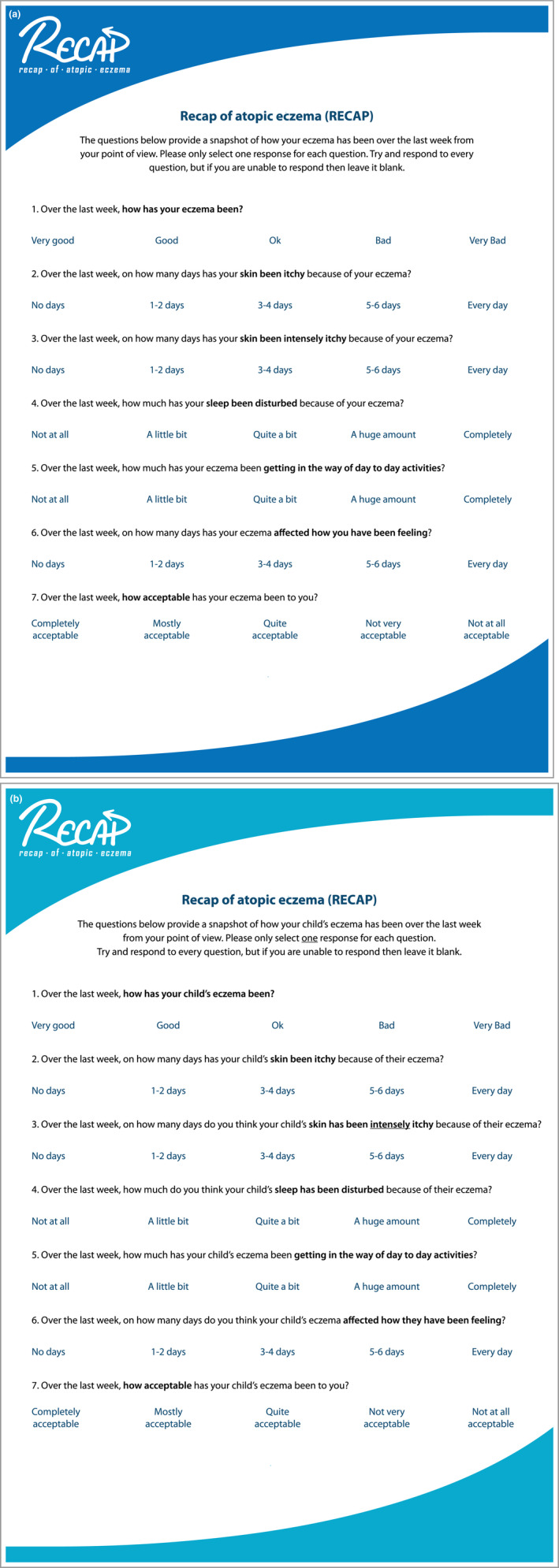
Recap of atopic eczema (RECAP) questionnaire (copyright retained by authors): (a) self‐reported version and (b) caregiver‐reported version.

The second model contained 10 items as it excluded ‘acceptability’ and ‘overall individual perception’ due to expert panel concerns that the more global nature of these items may remove important but more specific items. Appendix S4 and Table S3 (see Supporting information) show the full results of Model 2. The expert panel agreed Model 1 as the final set of items using the evidence from all previous stages of development. Model 1 was considered to be comprehensive and explained a larger proportion of the variance than Model 2 (Model 2: *R* ^*2*^ = 0·627, adjusted *R* ^*2*^ = 0·615, *F*(8, 256) = 53·74, *P* < 0·001). The final RECAP instrument can be found in Figure 4.

#### Stage 5: instrument scoring and preliminary validation

**Figure 5:**
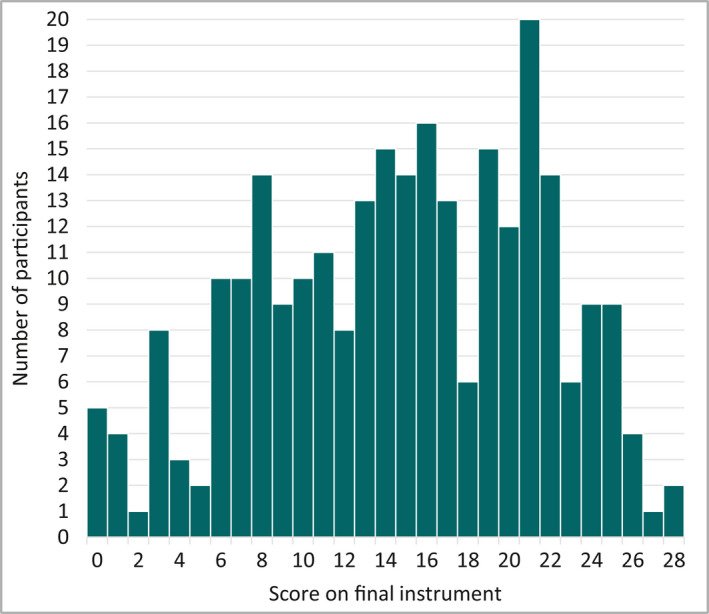
Distribution of scores on final instrument, *n* = 264.

Each of the seven questions in RECAP carries equal weight and is scored from 0 to 4 (total score of 0–28). The full scoring details are shown in Appendix S5 (see Supporting information). Figure 5 shows a normal distribution of scores and no floor or ceiling effects are present. The scores for the final instrument were significantly positively correlated with POEM scores, *r*(258) = 0·83, *P* < 0·001, which is in line with the hypothesis about convergence validity (construct validity). Table 7 illustrates how each increase in severity banding according to established POEM severity bandings and a single item global severity measure corresponded with a larger mean RECAP score for those scoring within that severity category,^25^ which is in line with hypotheses about discriminative validity (construct validity).

**Table 7:** Mean RECAP scores by severity categories

Severity categories	*n*	Mean (± SD)	Min–Max
POEM severity banding			
Clear/almost clear (0–2)	13	2·46 ± 2·67	0–7
Mild (3–7)	33	7·15 ± 3·99	0–19
Moderate (8–16)	94	12·64 ± 4·13	4–22
Severe (17–24)	94	18·72 ± 4·25	7–26
Very severe (25–28)	26	22·69 ± 3·18	14–28
Global severity response option^a^			
Clear	6	0·67 ± 1·21	0–3
Almost clear	34	6·88 ± 4·94	0–21
Mild	64	10·86 ± 4·10	3–20
Moderate	120	17·24 ± 4·50	6–26
Severe	39	22·15 ± 3·23	14–28

a ’How has your/your child's eczema been over the past week?’

POEM, Patient‐Oriented Eczema Measure; RECAP, Recap of atopic eczema.

## Discussion

RECAP is a patient‐ or caregiver‐reported instrument to capture ‘an individual's experience of eczema control’ intended for use in clinical trials and routine care. RECAP, comprising just seven questions, represents a practical and feasible approach to capturing a patient/caregiver's perspective of eczema control. The development process was designed to maximize the comprehensiveness, comprehensibility and relevance of the items to patients and caregivers while producing a tool that was feasible.^16^

How eczema control is conceptualized has implications for the most appropriate measurement model to use in developing RECAP. The study team engaged in multiple discussions about whether the construct of interest for RECAP was best considered as a reflective or a formative model. It was considered that each item was tapping into a different characteristic and contributing part of the construct, and when considered together they form the whole construct. Therefore, it was decided that a formative model was most appropriate. Furthermore, eczema control is a complex construct and therefore it was considered difficult to capture using only a single question, particularly as the term ‘control’ has multiple meanings in everyday language and can be interpreted in different ways.^27^

Given that eczema control includes a dimension of time, it was considered important to ask about experiences of eczema during a defined period rather than ‘at the moment’. It was initially felt that a 4‐week recall period may be a better indicator of ‘long‐term’ control. However, the 4‐week period used at the start of the study was found to affect the ability of patients to calculate a response due to difficulties with recall and if their eczema had varied greatly over that period of time, averaging out their experience.^28^ The chosen recall period of ‘the last week’ is in line with US Food and Drug Administration guidance, which states a preference for items that ask patients to describe their current or recent state.^8^

Regarding strengths and limitations, this instrument was purposefully developed so that it could be applied across all age groups and a self‐report and caregiver‐report version have been developed simultaneously to create a measure that will work across all trial populations. This quick‐to‐complete instrument could be transferred easily to online or smartphone application platforms. The questionnaire is free to access and use.

It may be that there are some differences in the ways that individuals who have eczema and caregivers perceive eczema control, which further research should explore. The initial development phase involved testing of the instrument in a UK population and in the English language only due to resources available. However, involvement of stakeholders across different countries in the development team was utilized to try and anticipate any difficulties in adaptation and translation that could be foreseen by the team. The recruitment methods were varied to try and reach different audiences. However, it is possible that there are potential biases in the types of people who would be willing to take part in focus groups, interviews and online surveys voluntarily.

In conclusion, RECAP is a new instrument to capture ‘eczema control’ over the past week. It was developed according to best practice for the development of patient‐reported outcome measures. Further studies are now required to confirm the psychometric properties of the RECAP instrument in different populations and to confirm the suitability of RECAP for use in research studies and clinical practice.

## Supporting information


**Appendix S1** Focus group topic guide.
**Appendix S2** Interview guide.
**Appendix S3** Detailed analysis of cognitive interviews.
**Appendix S4** Multivariable regression analysis: Model 2 (an alternative model that was not chosen by the expert panel).
**Appendix S5** Scoring instructions for Recap of atopic eczema (RECAP).
**Table S1** Cognitive interview coding framework (inductive coding in green text) (part of Appendix S3).
**Table S2** Documentation of changes between interview rounds (part of Appendix S3).
**Table S3** Model 2 Final output (part of Appendix S4).
