# Supplementary material for: Development and initial testing of a new instrument to measure the experience of eczema control in adults and children: Recap of atopic eczema (RECAP)
Source: Br J Dermatol. 2020 Feb 20;183(3):524–36. doi: 10.1111/bjd.18780 (PMC7496132; doi:10.1111/bjd.18780)
Supplement: Supplementary file 1 — Appendix S1 Focus group topic guide. Appendix S2 Interview guide. Appendix S3 Detailed analysis of cognitive interviews. Appendix S4 Multivariable regression analysis: Model 2 (an alternative model that was not chosen by the expert panel). Appendix S5 Scoring instructions for Recap of atopic eczema (RECAP). Table S1 Cognitive interview coding framework (inductive coding in green text) (part of Appendix S3). Table S2 Documentation of changes between interview rounds (part of Appendix S3). Table S3 Model 2 Final output (part of Appendix S4). [file BJD-183-524-s001.docx]

**Appendix S1. Focus group topic guide**

# Preparatory steps:

1. Ask everybody to fill out written consent forms
2. Turn tape recorder on
3. Explain the purpose of the session (to understand their individual experience of eczema control, to develop a way of measuring eczema control using a patient / parent reported questionnaire to be used in research studies, for individuals to use at home and to share with doctors at the clinic)
4. Explain ground rules: confidentiality of all participants and respect for all points of view (no right or wrong answers either, it is about your own experience and perspective)
5. Hand out name badges and ask to add their name or nickname

# Introductions

Take it in turns around the room providing a name they would like to be identified by in the session, whether they have eczema or have a child with eczema (or both) and share something about themselves they would be happy to share with the group (i.e. how long they have had eczema)

# Main discussion

Open questions:

1. If I was to ask you if your eczema has been well controlled what would you say? – ask everyone in the group to share this in turn
2. What were you thinking about when you were deciding if the eczema has been well controlled? – open discussion
3. What do you think is important to consider to understand if your eczema is well controlled? – open discussion
4. Over what time period where you thinking about when answering the previous questions? (Probes: e.g. over the last week, over the last day, over the last year etc.)

(Throughout questions 1-3, the co-facilitators will be taking notes)

REFRESHMENT BREAK

Discussion of the conceptual model previously developed:

1. Summarise what was discussed before the break and introduce/show the conceptual model of eczema control previously developed.
2. Probe discussion on comparison between what was identified by the focus group and the conceptual model, if any differences between these how this could be addressed and what may be missing from the conceptual model.

# Debrief

- Thank you to participants
- If any distress or medical concerns contact GP / Derm and tell about support groups available.
- Info on how to claim expenses

**Appendix S2. Interview guide**

**What are you thinking when you answer questions about your / your child’s eczema control?**

**Introductions**

- Greetings with participant/s
- If they have not already done so, ask participants to read information sheet and provide informed consent (for child as well if they are present)
- Give participants the opportunity to ask questions
- Explain the purpose of today’s interview is to help us make sure the questions we ask in eczema research and clinical practice are meaningful to people. We want to know how easy the questions are to understand, what the questions mean to you and what they make you think of.
- Check they are happy for the audio recording to be turned on and turn it on.
- Give participants an opportunity to share some information about themselves (listen out for age, sex, ethnicity, disease duration, and severity related information).
- Give them an option to have a practice at “thinking out loud” if they would like. If they would like to ask them to "Try to visualize the place where you live, and think about how many windows there are in that place. As you count up the windows, tell me what you are seeing and thinking about." (can probe if needed)
- Explain that you are going to ask them to fill out a questionnaire measuring eczema control (N.B. the exact questions will be developed in stage 1 but will ask about how various aspects of their eczema have been over a recent time period). If also going to be asking about the Patient Oriented Eczema Measure (POEM), explain that you are going to ask about this questionnaire on eczema symptoms too. When they answer the questions, they will need to “think out loud” as they are filling them in. The interviewer or the participant can read the question out loud too if this is preferable. Explain that you may also ask them some questions about what they are thinking throughout the interview. Explain that there are no right or wrong answers.

Prompts that can be used are presented below. These have been informed by cognitive theory of self-report questionnaires and some of them have been adapted from different sources (Thorneloe et al., 2017, Willis and Artino Jr, 2013, Willis, 1999, Irwin et al., 2009, Collins, 2003, Jobe and Herrmann, 1996). They are categorised according to the purpose of the prompt. Other probes in response to what participants say may also be used.

**To determine understanding/comprehension:**

- What does the term “INSERT TERM” mean to you?
- What did you understand by the term “INSERT TERM”?
- Does “INSERT TERM” used in this question sound OK to you, or would you say something different?
- What were you thinking about when answering that question?

**To determine how the respondent interprets the task demands of the question:**

- What is this question asking?
- Did you think questions (insert number) were asking about the same thing or something different?

**To determine relevance for this person:**

- Is this something you would consider as being relevant to your child’s eczema?
- Do you feel this question applies to your child?

**To determine ability to retrieve information:**

- What time period where you thinking about when you answered that question?
- What were you thinking about when answering that question?
- How did you remember that?
- Did you have a particular time period in mind when you were answering that question?

**To determine how the respondent reconstructs information to form an answer:**

- How did you manage to calculate that answer?
- How did you pull all of the information you had together to arrive at that answer?
- How did you arrive at that thought?
- How did you come up with your answer?

**To determine how confident respondent is in their judgement:**

- How easy or hard was it to come up with your answer?
- How sure are you about your answer?
- How well do you remember this?

**To understand why they chose a response option:**

- How did you decide on this answer?
- How did you decide to circle/tick that response?
- How well does the response that you circled/ticked apply to you?
- What do you think of the response choices here?
- Can you tell me why you chose the category “INSERT” rather than the category “INSERT”?
- Can you tell me why you chose to circle the category “INSERT” for this item, but you circled “INSERT” for this item?
- Did you change your mind at all during deciding what answer to pick?
- Did you have any difficulty deciding which response option to pick?
- Is there anything else you would have liked to have responded with that wasn’t here as an option?

**To encourage elaboration of “think aloud” process:**

- What made you say that?
- You were hesitating then – what were you thinking about?
- Is there anything else that you were thinking about?

**Overall questionnaire:**

- Are there any questions that you think don’t belong in this group?
- Are there things that we forgot to ask about that you think are important?
- What are your overall thoughts of these questions?
- Is there anything about the look of the questions that you don’t like?
- Is there anything else you would want to change about these questions?
- Give the opportunity to add anything else they would like to say.
- Debrief – Reiterate the purpose of this study. If any concerns have been raised by the participant, provide information of where they could receive further support (GP, National Eczema Society – www.eczema.org)

Appendix S3. Detailed analysis of cognitive interviews

Data were analysed using a problem-focused approach using a hybrid model that used a top-down framework that coded for both problems relating to cognitive processes and question features (Table S1). However, this framework could be refined using inductive coding (inductive codes added are highlighted in green).

Table S1. Cognitive interview coding framework (inductive coding in green text)

| **Code** | **Label** | **Elaboration** |
| --- | --- | --- |
| **1** | **Comprehension** | Item has ambiguous meaning, lack of clarity in wording, uses obscure or difficult language |
| **2** | **Intended construct** | Raised a concern about if participant is responding in a way that is capturing the intended construct |
| **2.1** | **Beliefs about their eczema and/or treatments affecting response** | i.e. related to eczema but not the concept we are trying to capture |
| **2.2** | **None eczema related issues affecting response** | i.e. not related to the eczema, other diseases, other reasons |
| **3** | **Knowledge** | Participant lacked the information needed to answer the question |
| **4** | **Applicability** | Item was not relevant or applicable to the participant, question had made  assumptions |
| **5** | **Sensitivity / Desirability** | Item raised concerns or wording was too sensitive, desirability bias likely to occur |
| **6** | **Memory retrieval** | Participant had difficulty recalling information required, high level of detail required, recall period too long, felt they had a shortage of cues |
| **7** | **Calculating response** | Participant had to make a complex estimation to decide upon a judgement or evaluation, had to use heuristics to provide answer |
| **8** | **Assigning response options** | Response options were undefined or vague, used inappropriate units, unclear what they referred to, overlapping categories, missing categories |
| **8.1** | **Distinguishing between response option types** | i.e. not clearly distinguishing frequency response options from intensity response options |
| **9** | **Other concerns raised** | Problems identified that do not fit within the above codes |
| **9.1** | **Aim of the questions** | Uncertainty about the aims of the questions |
| **9.2** | **Uncertainties when making comparisons** | Uncertainties about what experience to compare current experience to |

Table S2 (below) illustrates the changes that took place between four rounds of cognitive interviews. The results are detailed below, but to summarise, the recall period was changed from 4 weeks to 1 week, the response options were changed from 4 to 5, the items were changed from statements to questions, wording was changed to provide clarity, and language was amended to reflect terms that felt more resonant to respondents and increase the confidence of respondents in their ability to answer the questions. By the end of the interviews 15 items remained for further testing.

**Changes to recall period**

Participants found it difficult to remember the recall period throughout answering the questions, as well as finding 4 weeks a long time to recall their experience. Therefore, following round 1 the recall period was stated in each question and reduced to 1 week.

“So the first thing I have to think about is if it’s four weeks where does that four weeks start from, and what’s been happening in my life at that time, and can I remember what my skin’s been doing in that last four weeks, and that’s not necessarily an easy thing, it takes quite a lot of thought to try and work all of that out.” (Elsa, person with eczema)

**Changes to response options**

Participants felt that the 4-point rating scales did not cover the full range of experience, expressing a desire to answer in a way that fell between two of the response options. Therefore, 5-point rating scales that aimed to more finely capture the full continuum of experience were presented in round 2 onwards.

“I suppose, this is one way it would be helpful to probable just have a rarely because I wouldn’t go as far as to say some of the time, but there have been on one or two occasions, where he’s just really, really scratching. Where eczema is… and we always kind of distract him from scratching himself. So, but I’d say that’s probably been once or twice over the past four weeks and yes, he does seem distracted from what he’s doing before because he suddenly wants to scratch and scratch and scratch. So, it’s closest to none of the time, some of the time because it literally only… I don’t know. Some in my mine, is I would expect it to be once or twice a week over the past four weeks, but yes. Can I write rarely on it?” (Natalie, caregiver)

Challenges with multiple possible interpretations of the response options presented as a ‘proportion of the time’ were addressed by changing to number of days.

“So, I guess, all of the time is every hour of every day. And most of the time would be many occasions through the day with some of the time I think maybe just one or two things in a day. Then it’s hard to know the severity. So, overnight he’s been waking once or twice normally, so that’s not all the time. But that is every night. So, that’s hard to know.” (Leslie, caregiver)

Whilst still using a 4-week recall period the number of days response options remained as descriptors rather than specifying a number of days, which still required difficult calculations for respondents, as they were trying to convert the “vague” descriptors within the response options into how they would interpret how many days in the last 4 weeks they felt that described. Since the version developed for round 3 had a recall period of one week, it was possible to alter the response options to be more specific within the description what range of days were included in each response category.

**Change from statements to questions**

Using statements for items did not match the ‘number of days’ response options. Participants in round 1 also felt the statements led to a desire to respond in a ‘yes/no’ format, whereas the developers wanted to measure each item as a range of experience. Therefore, the items were changed into a question format.

**Removing and adding items**

Entering the cognitive-interview phase, the expert panel had included more than one item that tapped onto each important concept within the conceptual model, to help determine which way of asking about a particular construct would be most appropriate in this setting. Some of the items were judged as not being easily interpreted or as clearly aligned with the intended construct as others, and therefore removed during the interview rounds, and in some cases additional items were added as new ways to approach capturing the concepts.

There was one area where the removal of items resulted in the refinement of the conceptual model. Four items were developed that were intended to capture constructs relating to the treatment and management of the condition. However, in designing and testing the items it became clear that it was difficult to establish items that would be applicable across all populations and for all types of clinical trials. Therefore, all treatment questions were removed at this stage except for one which was adjusted into a global question about the impact of treatment on control to improve applicability.

**Changes to language used in items**

The term “control” (and its derivatives) was thought to be a term that may be interpreted differently by all. The developers initially felt rephrasing to give permission for respondents to use their own interpretation may be sufficient, given that the experience of control was conceptualised as an individual experience within the conceptual framework. However, further exploration made it apparent that there were multiple interpretations of the term and some were not in line with the construct of interest. Therefore, it was decided that the term ‘control’ should be avoided in all items.

Well, I’d say that to you, so control of eczema that you manage it, so that you’ve done all your creams, you’ve done all your lotions, you’ve taken whatever tablets you take, so you’ve followed your regime to manage your eczema or is it about say, managing it so that it’s clear so – I’m not clear what you’re asking so, that’s why I say it depends on whether it’s control and management or if its clearing it up. So, if it’s clearing it up the answer is no days because it never goes. If it’s control and management inline of what I’ve to do, then it’s every day. (Amy, person with eczema)

There were other changes in the language used designed to provide clarity, use language that felt more resonant to respondents and increase the confidence of respondents in their ability to use their knowledge to answer the question.

**Table S2. Documentation of changes between interview rounds**

| Part of questionnaire | Round 1 | Round 2 | Round 3 | Round 4 |
| --- | --- | --- | --- | --- |
| **Recall period** | 4 weeks (in instructions only) | 4 weeks (in instructions and questions) | 1 week (in instructions and questions) | 1 week (in instructions and questions) |
| **Number of response options per item** | 4 | 5 | 5 | 5 |
| **Response option wording** | None of the time / Some of the time / Most of the time / all of the time | No days / Hardly any days / Some days / Most days / Every day | No days / 1-2 days / 3-4 days / 5-6 days / Every day | No days / 1-2 days / 3-4 days / 5-6 days / Every day |
|  | Not at all / A little / A lot / Completely | Not at all / A little bit / Quite a lot / A huge amount / Completely | Not at all / A little bit / Quite a lot / A huge amount / Completely | Not at all / A little bit / Quite a lot / A huge amount / Completely |
|  |  |  | Not at all controlled / A little controlled / Quite controlled / Mostly controlled / Completely Controlled | Very good / Good / OK / Bad / Very bad |
|  |  |  | Not at all acceptable / Not very acceptable / Quiet acceptable / Mostly acceptable / Completely acceptable | Completely acceptable / Mostly acceptable / Quite acceptable / Not very acceptable / Not at all acceptable |
| **Item wording that was refined** | Overall, my eczema has been well controlled. | On how many days in the last 4 weeks would you describe your eczema as well controlled? | Over the last week, how would you describe your eczema? / Over the last week, on how many days would you describe your eczema as having been well controlled? | Over the last week, how has your eczema been? |
|  | My level of eczema control has been acceptable to me. | On how many days in the last 4 weeks has your level of eczema control been acceptable to you? | Over the last week, how acceptable has your level of eczema control been to you? / Over the last week, on how many days has your level of eczema control been acceptable to you? | Over the last week, how acceptable has your eczema been to you? |
|  | My skin has been itching because of my eczema. | On how many days in the last 4 weeks has your skin been itching because of your eczema? | Over the last week, on how many days has your skin been itchy because of your eczema? | Over the last week, on how many days has your skin been itchy because of your eczema? |
|  | My skin has felt painful/sore because of my eczema. | On how many days in the last 4 weeks has your skin felt painful/sore because of your eczema? | Over the last week, on how many days has your skin felt painful or sore because of your eczema? | Over the last week, on how many days has your skin felt painful or sore because of your eczema? |
|  | I have been experiencing eczema symptoms. | On how many days in the last 4 weeks have you experienced at least one eczema symptom? | Over the last week, on how many days have you had any signs or symptoms of your eczema? | Over the last week, on how many days have you had any symptoms of your eczema? |
|  | I have been experiencing eczema flares. | On how many days in the last 4 weeks have you been experiencing eczema flares? | Over the last week, on how many days have you experienced an eczema flare? | Over the last week, on how many days have you experienced an eczema flare? |
|  | I have felt isolated because of my eczema. | On how many days in the last 4 weeks have you felt isolated because of your eczema? | Over the last week, on how many days have you felt isolated because of your eczema? | Over the last week, on how many days have you felt isolated because of your eczema? |
|  | I have felt embarrassed because of my eczema. | On how many days in the last 4 weeks have you felt self-conscious or embarrassed because of your eczema? | Over the last week, on how many days have you felt self-conscious or embarrassed because of your eczema? | Over the last week, on how many days have you felt self-conscious or embarrassed because of your eczema? |
|  | My eczema has affected how I have been feeling. | On how many days in the last 4 weeks has your eczema affected how you have been feeling? | Over the last week, on how many days has your eczema affected how you have been feeling? | Over the last week, on how many days has your eczema affected how you have been feeling? |
|  | My eczema has been getting in the way of my everyday life. | In the last 4 weeks, how much has your eczema been getting in the way of your everyday life? | Over the last week, how much has your eczema been getting in the way of your day to day activities? | Over the last week, how much has your eczema been getting in the way of your day to day activities? |
|  | My sleep has been disturbed because of my eczema. | In the last 4 weeks, how much has your sleep been disturbed because of your eczema? | Over the last week, how much has your sleep been disturbed because of your eczema? | Over the last week, how much has your sleep been disturbed because of your eczema? |
|  | My eczema treatment has been enough to control my eczema. | Thinking about all the eczema treatments you have used in the last 4 weeks, on how many days has your treatment been enough to control your eczema? | Thinking about all the eczema treatments you have used in the last week, on how many days has your treatment been enough to control your eczema? | Thinking about all the eczema treatments you have used in the last week, on how many days has your treatment been enough to manage your eczema? |
| **Items that were added** |  |  |  | Over the last week, on how many days were you unable to stop scratching? |
|  |  |  | Over the last week, on how many days has your skin felt intensely itchy because of your eczema? | Over the last week, on how many days has your skin felt intensely itchy because of your eczema? |
|  |  | On how many days in the last 4 weeks has your eczema stopped you doing something you wanted or needed to do? | Over the last week, on how many days has your eczema stopped you doing something you wanted or needed to do? | Over the last week, on how many days has your eczema stopped you doing something you wanted or needed to do? |
| **Initial items that were removed before the final round** | My life has been impacted by my eczema. | In the last 4 weeks, how much has your life been impacted by your eczema? | Over the last week, how much has your life been affected by your eczema? |  |
|  | I have been distracted because of my eczema. | On how many days in the last 4 weeks have you been distracted because of your eczema? | Over the last week, on how many days have you been distracted because of your eczema? |  |
|  | Overall, my eczema has been controlled. | On how many days in the last 4 weeks would you describe your eczema as under control? |  |  |
|  | I have modified my everyday life because of my eczema. | In the last 4 weeks, how much have you modified your everyday life because of the eczema? |  |  |
|  | I have been able to control my eczema. |  |  |  |
|  | I have been affected by my eczema symptoms. |  |  |  |
|  | I have been affected by eczema flares. |  |  |  |
|  | My eczema has felt unpredictable. |  |  |  |
|  | My eczema has affected my appearance. |  |  |  |
|  | My eczema has been on my mind. |  |  |  |
|  | I feel able to manage my eczema using the treatment I have. |  |  |  |
|  | The amount of time I have spent treating my eczema troubles me. |  |  |  |
|  | My eczema treatment has not been enough to control my eczema. |  |  |  |

*Note.* The self-reported version is reported here for brevity, but the same changes were made for the caregiver-reported version

**Appendix S4. Multivariable regression analysis: Model 2 (an alternative model that was not chosen by the expert panel)**

The second model contained 10 items entered into model 1 as predictor variables, with the exclusion of ‘acceptability of eczema’ and ‘global’ due to expert panel concerns that the more global nature of these items may be problematic for inclusion in the regression model with more specific items. The bother scale was used as the outcome variable.

Two predictor variables were removed from the model following a backward elimination item reduction technique with a stopping criterion of *p* = 0.157. These included items ‘being unable to stop scratching’ (*p* = 0.808) and ‘painful or sore skin’ (*p* = 0.273).

The results of the regression indicated that the seven remaining predictor variables explained 61.5% of the variance in bother, *R^2^* = 0.627, adjusted *R^2^* = 0.615, *F*(8, 256) = 53.74, *p* < 0.001. Table 4‑7 shows the predictor variables that remained in the model.

Table S3 Model 2 Final Output

| Dependent Variables | β | P-value | 95% CI |
| --- | --- | --- | --- |
| Itchy skin | 0.32 | 0.007 | 0.09, 0.55 |
| Sleep disturbance | 0.26 | 0.016 | 0.05, 0.47 |
| Getting in the way of day to day activities | 0.64 | >0.001 | 0.33, 0.95 |
| Affecting how been feeling | 0.24 | 0.013 | 0.05, 0.43 |
| Stopped from doing something wanted or needed to do | -0.21 | 0.068 | -0.45, 0.02 |
| Having flares | 0.41 | >0.001 | 0.22, 0.60 |
| Having any symptoms | 0.20 | 0.069 | -0.02, 0.41 |
| Intensely itchy skin | 0.21 | 0.038 | 0.01, 0.41 |

**Appendix S5. Scoring instructions for RECAP**

Each of the seven questions carries equal weight and is scored from 0 to 4:

| Very good  Good  OK  Bad  Very bad | =0  =1  =2  =3  =4 | No days  1-2 days  3-4 days  5-6 days  Every day | =0  =1  =2  =3  =4 | Not at all  A little bit  Quite a lot  A huge amount  Completely | =0  =1  =2  =3  =4 | Completely acceptable  Mostly acceptable  Quite acceptable  Not very acceptable  Not at all acceptable | =0  =1  =2  =3  =4 |
| --- | --- | --- | --- | --- | --- | --- | --- |

**Note:**

- If one question is left unanswered this is scored 0 and the scores are summed and expressed as usual out of a maximum of 28.
- If two or more questions are left unanswered the questionnaire is not scored.
- If two or more response options are selected, the response option with the highest score should be recorded.

*The assumptions made for these scoring rules are outlined below:*

1. *If one question is left unanswered, it is assumed that the item is not of relevance to the individual’s control, and it is considered most appropriate to give ‘no relevance’ a score of zero.*
2. *If two or more questions are left unanswered, it is assumed that there is a potential problem with filling out the questionnaire, and it is regarded as too much missing data within the items to generate a total score.*
3. *If two more responses are selected in one item, it is considered preferable to try and provide a score and reduce missing data, and the response option with the highest score is chosen as it is assumed this will reduce the likelihood of underestimating the impact of eczema on the individual.*
